# Real-Time Cellular Exometabolome Analysis with a Microfluidic-Mass Spectrometry Platform

**DOI:** 10.1371/journal.pone.0117685

**Published:** 2015-02-27

**Authors:** Christina C. Marasco, Jeffrey R. Enders, Kevin T. Seale, John A. McLean, John P. Wikswo

**Affiliations:** 1 Vanderbilt Institute for Integrative Biosystems Research and Education, Vanderbilt University, Nashville, Tennessee, United States of America; 2 Department of Biomedical Engineering, Vanderbilt University, Nashville, Tennessee, United States of America; 3 Department of Chemistry, Vanderbilt University, Nashville, Tennessee, United States of America; 4 Vanderbilt Institute of Chemical Biology, Vanderbilt University, Nashville, Tennessee, United States of America; 5 Department of Physics and Astronomy, Vanderbilt University, Nashville, Tennessee, United States of America; 6 Department of Molecular Physiology and Biophysics, Vanderbilt University School of Medicine, Nashville, Tennessee, United States of America; George Mason University, UNITED STATES

## Abstract

To address the challenges of tracking the multitude of signaling molecules and metabolites that is the basis of biological complexity, we describe a strategy to expand the analytical techniques for dynamic systems biology. Using microfluidics, online desalting, and mass spectrometry technologies, we constructed and validated a platform well suited for sampling the cellular microenvironment with high temporal resolution. Our platform achieves success in: automated cellular stimulation and microenvironment control; reduced non-specific adsorption to polydimethylsiloxane due to surface passivation; real-time online sample collection; near real-time sample preparation for salt removal; and real-time online mass spectrometry. When compared against the benchmark of “in-culture” experiments combined with ultraperformance liquid chromatography-electrospray ionization-ion mobility-mass spectrometry (UPLC-ESI-IM-MS), our platform alleviates the volume challenge issues caused by dilution of autocrine and paracrine signaling and dramatically reduces sample preparation and data collection time, while reducing undesirable external influence from various manual methods of manipulating cells and media (*e.g.*, cell centrifugation). To validate this system biologically, we focused on cellular responses of Jurkat T cells to microenvironmental stimuli. Application of these stimuli, in conjunction with the cell’s metabolic processes, results in changes in consumption of nutrients and secretion of biomolecules (collectively, the exometabolome), which enable communication with other cells or tissues and elimination of waste. Naïve and experienced T-cell metabolism of cocaine is used as an exemplary system to confirm the platform’s capability, highlight its potential for metabolite discovery applications, and explore immunological memory of T-cell drug exposure. Our platform proved capable of detecting metabolomic variations between naïve and experienced Jurkat T cells and highlights the dynamics of the exometabolome over time. Upregulation of the cocaine metabolite, benzoylecgonine, was noted in experienced T cells, indicating potential cellular memory of cocaine exposure. These metabolomics distinctions were absent from the analogous, traditional “in-culture” UPLC-ESI-IM-MS experiment, further demonstrating this platform’s capabilities.

## Introduction

Cellular response is a complex phenomenon that manifests both physically and chemically. While physical responses can most often be analyzed visually, chemical responses are difficult to characterize even with modern detection methods. Adding to the difficulty is the influence of timing when dealing with cellular response. When responding to a biochemically altering stimulus, such as naïve T-cell response to an antigen presented by a dendritic cell, a cell undergoes a series of biochemical pathway shifts that allow it to adapt to its new conditioned state. The cell’s temporal response, therefore, provides insight into the interspersed pathway shifts that occur throughout the various stages of stimulus exposure. Temporal response is especially relevant to toxicology, where, despite experiencing a toxin and undergoing numerous metabolic shifts, an organism may not show observable external symptoms until much later, when treatments may be less effective.

Nonetheless, time remains a largely underappreciated or neglected variable in most comprehensive cellular response experiments, not as much for its perceived lack of value as for the difficulty of precise temporal resolution and control of measurements. Biological measurements typically take one of two forms; either they sample a small number of targets very frequently (as in immunoassay or fluorescence measurements) or they sample a large number of targets over a broad time span (*e*.*g*., proteomic cataloging). The limitations of current methodological strategies and detection technologies hinder the combination of these two approaches. The most comprehensive analysis would track as many analytes as possible and sample these analytes as frequently as possible to capture the truly dynamic properties of metabolic responses. As the frequency of sample collection is increased, previously unidentified patterns in signal response may begin to emerge, according to the Nyquist-Shannon sampling theorem, which states that in order to properly characterize a pattern that has as its highest frequency Y, the sampling must occur at intervals less than 1/(2Y), thereby eliminating the possibility of aliasing, wherein undersampling of rapidly varying phenomena produces misleading features that appear to have a lower frequency.

Given the desire to study dynamic biological phenomena within the construct of robust microphysiological systems [1], a major logistical problem still remains: how does one sample a biological system multiple times over the course of an experiment without destroying, or even perturbing, that system? The exometabolome, or suite of biomolecules that are secreted or excreted from a cell into the surrounding matrix, represents a promising target. The exometabolome has been demonstrated as a viable indicator of internal cellular processes and can be easily assessed without any disturbance of the system from which it originates [2]. Changes in the transport of exometabolomic species provide information on the current state of the cell population, which can lead to a more thorough understanding of the particular cell biology and to the ability to control cell behavior [3]. Further promoting the benefit of metabolomics is the timescale of metabolic response to alterations in the environmental conditions. While alterations in the proteome occur over hours or days, metabolome changes occur within seconds or minutes.

The cell is constantly surveying the surrounding environment. Perceived changes in this microenvironment lead to alterations of intracellular and intercellular signaling, which in turn lead to shifts in gene regulation and modifications in protein and metabolite production. Depending on the signal received, the intracellular processes enacted may lead to the secretion of another signaling factor to extend the complex web of communication. These signaling factors are produced by a given cell for communication with 1) nearby cells of the same type (autocrine signaling), 2) adjoining cells (juxtacrine signaling), 3) nearby cells of a different type (paracrine signaling), and 4) distant cells (endocrine signaling). The exometabolome not only includes traditional signaling molecules and metabolites of nutrients, but also enzymatically produced metabolites of xenobiotics (*i*.*e*., drugs, toxins) introduced to cells and the nutrients or other factors in the culture media as modified through cellular uptake.

The Vanderbilt Institute for Integrative Biosystems Research and Education Multitrap Nanophysiometer (MTNP) is a polydimethylsiloxane (PDMS) microfluidic device that functions as a miniature bioreactor for unattached cells [4–10]. In contrast to traditional cell biology techniques that often require cellular populations on the order of >10^8^, the MTNP allows for studies on small populations of cells or simple organisms (<10^5^). The MTNP also provides a solution to the volume challenge problem existing in traditional in-culture experiments, as the small volume of the device and continuous flow prevent the dilution of signaling factors [7]. The MTNP can be used for long-term optical measurements of the dynamic behavior of cells, including fluorescent labeling of cells to determine type and activation state and detect signaling dynamics and cell-cell interaction. This device provides a framework on which to study numerous cells, *i*.*e*., T cells, beta cells, and breast cancer cells, as it traps both non-adherent and adherent cells with structural barriers instead of with chemical surface modification that may cause cells to be exposed to higher shear stresses resulting from direct contact with fluid flow. The MTNP is well suited to detect secreted molecules in cellular effluent, and it is also unique in its ability to provide a system for investigating the dynamic response of a cell population to a stimulus, possibly enabling challenge-response statistical analysis of cellular dynamics [11]. The constant perfusion design of the microfluidic bioreactor gives rise to a platform component capable of real-time alteration and control of the cellular microenvironment, in addition to providing an opportunity for monitoring the effluent output from the device.

The complexity of biological samples often overwhelms standard screening techniques seeking to discover biomarkers of disease or rapidly assess drug efficacy. This unique problem warrants an analytical technique capable of both rapid screening of samples and sufficient sensitivity to account for the many analytes in low abundance that typically are of interest in metabolomic and signaling experiments, which often either defy detection or appear collectively as chemical noise. The use of mass spectrometry (MS) as the leading detection method of proteomic [12,13], lipidomic [14], and glycomic [15] studies has led to many advances in elucidating the complexity of the cell.

The combination of constant microfluidic perfusion bioreactors with mass spectrometry has the potential to rapidly screen cell effluent for secreted species indicative of internal metabolic perturbations. This is potentially of great importance for analyzing the response of coupled organ-on-chip systems to drugs, toxins, and other agents [1,16,17]. Several studies have taken on the challenge of integrating these technologies to produce a powerful analytical platform. In an early experiment, Chan *et al*. verified that using PDMS bioreactor devices to transfer samples into the electrospray ionization mass spectrometer (ESI-MS) did not contaminate the samples [18]. On-chip ultrafiltration and analyte pre-concentration for high-throughput small molecule screening with ESI-MS were performed with the resulting detection sensitivity shown to increase by one to two orders of magnitude over off-chip screening strategies [19]. A significant impairment in coupling cellular bioreactor microfluidic devices with online-MS is in the suppression of signals of interest by salts present in biological samples. To overcome this challenge, some form of desalting is typically performed offline, using either a solid phase extraction (SPE) column and vacuum manifold or some form of liquid chromatography. These techniques desalt samples by providing a functionalized surface for which salts and analytes have differing affinities. For example, liquid chromatography (LC) typically uses C18 columns, which provide binding sites for non-polar molecules but lack sufficient interaction prospects for salts, thereby allowing for an aqueous rinse to clear the column of salts and a subsequent organic elution of analytes without the suppressing contributions from salts. These methods, while providing an efficient means of desalination, come at the expense of temporal resolution, and they are not designed for online analysis. One recent development in the online desalting of effluent from a microfluidic bioreactor is the work of Chen *et al*., which incorporates a packed micro-solid phase extraction column [20].

We have previously demonstrated the ability to rapidly desalt a continuous sample stream using online SPE [21,22]. This work directly translates to the processing of continuous sample streams originating from an MTNP. Using online SPE for such applications provides a surface for which analytes of interest have affinities but unwanted salts do not. This allows for variable duration periods of pre-concentration of the analytes and even the potential for gradient elution of these analytes.

**Fig 1:**
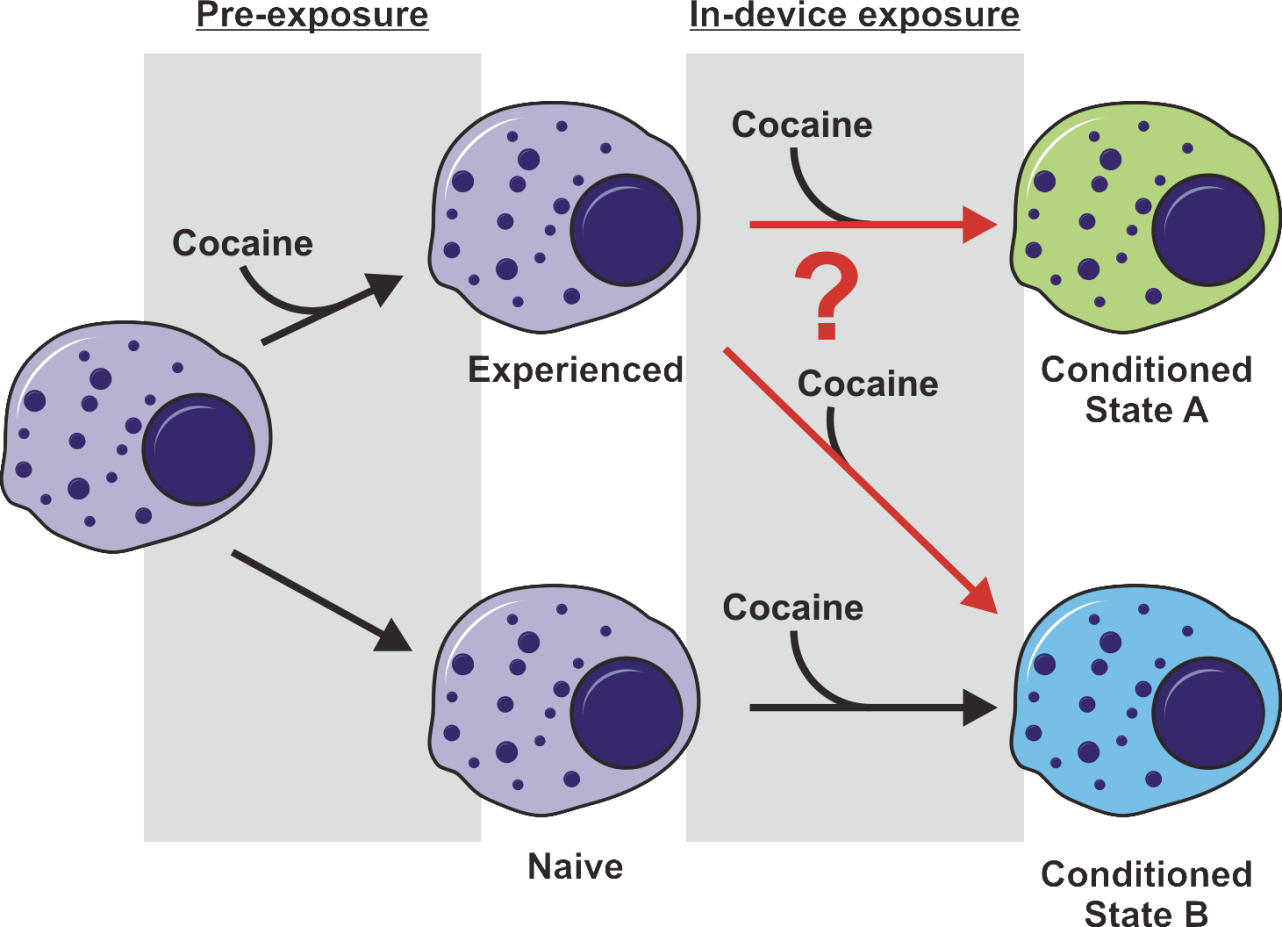
Experimental scheme showing potential cell fates. Upon exposure to cocaine in a microfluidic bioreactor, naïve and cocaine-experienced cells present different exometabolomic profiles, demonstrated here as color change. Our experiments were designed to determine whether cocaine-experienced cells went to a conditioned state A that was different from state B reached by naïve cells.

As an initial proof of concept experiment for this integrated platform, we selected cellular memory of drug exposure, specifically Jurkat T-cell memory of cocaine, for ease in identification of known drug metabolites and for its unique biological information. The choice of cell type was based upon cocaine’s classification as an immunosuppressive agent, by mechanisms of direct and/or indirect actions on immune cells. Findings from experiments tying cocaine to immune function suppression have been contradictory [23–27], most likely because of the complex biological systems under investigation and wide disparity of experimental procedures. A major hurdle in determining these mechanisms is the lack of an assay capable of tightly controlled environmental parameters, sufficient temporal resolution to avoid loss of transient changes, and multi-parameter sampling for unique evidence of interconnection of experimental variables. The platform demonstrated herein allows for such an assay to be conducted. Through the comparison of naïve Jurkat T cells and those with prior cocaine exposure on this platform, differences in cocaine metabolism are detected. Fig. 1 demonstrates the experimental concept. Exposure of a naïve cell to cocaine may lead to a certain dynamic exometabolomic profile that defines the state of the cell. However, when a cell with prior cocaine experience receives a subsequent dose, the dynamic exometabolomic profile may either be identical to that of the naïve cell under exposure or distinguish the cell as unique. With a near real-time readout of cellular metabolic events on our integrated microfluidic-solid phase extraction-ion mobility-mass spectrometry platform, it is possible to determine variations in dynamic exometabolomic profiles that will provide evidence of cellular memory of cocaine exposure.

## Methods

### Microfluidic Bioreactor Design and Fabrication

The Vanderbilt Institute for Integrative Biosystems Research and Education Multitrap Nanophysiometer was previously designed in AutoCAD and converted to a chrome mask on glass (Advance Reproductions, North Andover, MA). A soft-lithographic master was produced through photolithography methods, which include spinning a negative photoresist, SU-8, onto silicon wafers, exposing them to UV light through the desired mask to crosslink the SU-8, then developing to remove non-crosslinked polymer [28]. Polydimethylsiloxane (PDMS) (Sylgard 184 Elastomer Kit, Dow Corning, Midland, MI) was then cast onto the silicon and SU-8 master, cured, and removed from the master. Inlet and outlet ports were punched and PDMS replicas were bonded to glass coverslips by O_2_ plasma treatment (Harrick Plasma Cleaner, Ithaca, NY). PDMS surface modification was performed using alcohol deposition of 2-[methoxy(polyethyleneoxy)_6–9_propyl]trimethoxysilane immediately following plasma treatment and bonding (see Supporting Information, S1 File, S1 Fig.).

### Cell Culture and “In-Culture” Cocaine Exposure

Jurkat T cells (clone E6-1, TIB-152) (ATCC, Manassas, VA) were cultured in 90% RPMI 1640, 10% fetal bovine serum (FBS, heat inactivated) (Lonza, Allendale, NJ) at 37°C, 5% CO_2_ according to the manufacturer’s instructions.

**Fig 2:**
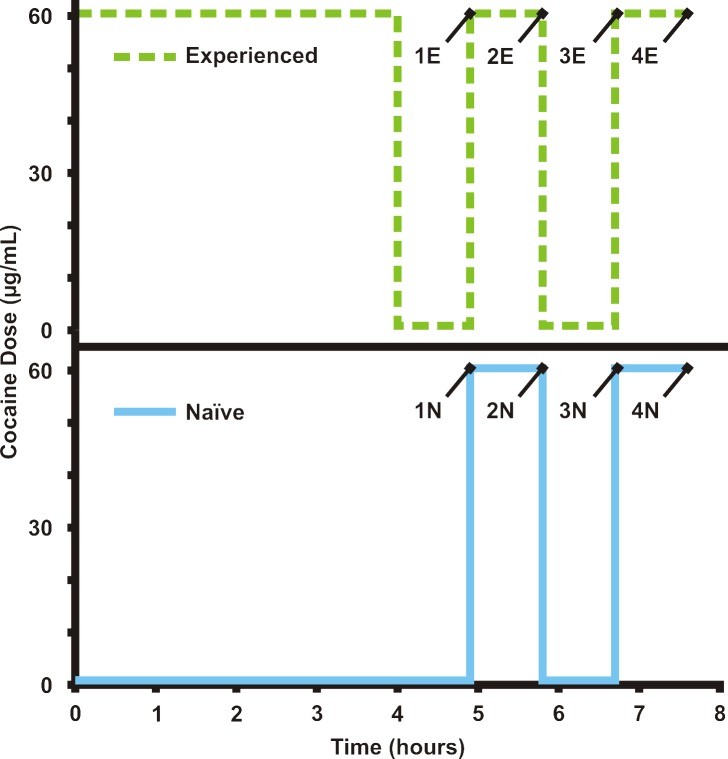
Cocaine exposure scheme for both in-culture and online cell experiments. The time course of cocaine administration to naïve (blue) and experienced (green) T-cell populations is shown. For the in-culture experiments, experienced samples 1E-4E and naïve samples 1N-4N were withdrawn for analysis at the times shown.

In-culture cocaine exposure experiments were performed as follows (Fig. 2). Two populations of Jurkat T cells (passage 6, 2 million cells/mL, 500 μL, in biological triplicate) from the same culture flask were added to two separate microcentrifuge tubes. Both tubes were centrifuged at 200 × g for 2 minutes and supernatant was removed. Cells in Tube 1 were resuspended in 500 μL of RPMI 1640 and incubated for 270 minutes at 37°C, 5% CO_2_. Cells in Tube 2 were resuspended in 500 μL of cocaine in RPMI 1640 (60 μg/mL) and incubated for 216 minutes at 37°C, 5% CO_2_. The cells in Tube 2 were centrifuged and resuspended in RPMI 1640 for 54 minutes. At this point, the cells in Tube 1 are “cocaine naïve,” while those in Tube 2 are “cocaine experienced.”

Both tubes were then centrifuged as above, with the supernatant being reserved as Samples 1E and 1N for protein precipitation and metabolomic analysis. The cell pellets were immediately resuspended in RPMI with cocaine at 60 μg/mL and incubated for another 54 minutes.

After this 54-minute cocaine exposure, the cells were centrifuged to obtain 2E and 2N, representing the results from the first common cocaine exposure for both naïve and experienced cells. The cells were resuspended in RPMI 1640 for another 54 minutes. Centrifugation provided samples 3E and 3N, representing recovery from cocaine exposure.

Next, the cells were resuspended with RPMI and cocaine (60 μg/mL) and incubated for 54 minutes. A final centrifugation provided samples 4E and 4N, representing the final cocaine exposure.

In order to investigate the degradation of cocaine over the time course of the experiment, samples of cocaine in RPMI but without cells were incubated in the same conditions as the cells and sampled at each 54-minute time point starting at time zero and ending at 10.5 hours.

### Metabolomics Sample Preparation and Analysis

As each 54-minute time point was taken, supernatant was placed on ice to halt any continued metabolite modifications and immediately processed for protein removal with cold methanol. To each 300 μL supernatant sample, 900 μL of cold methanol was added, vortexed, allowed to sit for 10 minutes at 4°C and then centrifuged at maximum speed for 10 minutes in a Heraeus Fresco 21 temperature-controlled centrifuge (Thermo Scientific). Supernatant was transferred to a new microcentrifuge tube and kept at 4°C until all samples were collected and processed. All samples were then dried down in a Savant DNA 110 Speedvac overnight with low heat and reconstituted in 300 μL 5% methanol/95% water (0.01% formic acid).

Samples were placed in the sample tray of the nanoAcquity UPLC with autosampler (Waters, Milford, MA), which remains cooled to 4°C to prevent sample degradation. Ultraperformance liquid chromatography (UPLC) was performed on 1 μL samples loaded on a HSS C18, 1.8 μm particle size column with mobile phase A (0.1% formic acid in H_2_O) by ramping from 100% mobile phase A to 100% mobile phase B (0.1% formic acid in MeOH) over 11 minutes, then holding at 100% mobile phase B for 2 minutes. Ion mobility-mass spectrometry and MS^e^ were then performed on the eluted analytes using an ion mobility-mass spectrometer (IM-MS, Synapt G2, Waters Corp., Milford, MA). Quality control samples were dispersed every 10 samples in the Waters MassLynx v4.1 software sample list among technical triplicates of each biological sample.

### Online Cell Loading and Experimentation

Prior to experimentation, 500–1000 μL of cell suspension was removed from culture flasks. Cells at passage 6 were used for experiments. Cells were then gently pelleted and aspirated into polyether ether ketone (PEEK) tubing connected to pump-controlled syringes. The flow direction of the pump was reversed upon intubation of the MTNP and cells were collected into microfluidic traps for experimentation. Cell-loaded devices were then perfused with selected media components and brightfield images were collected by an inverted Nikon Eclipse Ti-e (Nikon Instruments, Melville, NY). Cells in the MTNP were maintained at 37°C and 5% CO_2_ during experimentation.

For T-cell cocaine metabolism studies, populations of naïve T cells and experienced T cells (exposed to cocaine at 60 μg/mL in RPMI 1640 for 4 hours) were stimulated with either cocaine (60 μg/mL in RPMI 1640) or RPMI 1640. Cells were initially exposed to plain RPMI media for 54 minutes, followed by exposure to cocaine at 60 μg/mL in RPMI for 54 minutes. Both steps were repeated for a total of four steps. Experiments with naïve and experienced cells were performed on the same day, in series, to exclude any variation in cell population from day to day. The cocaine exposure scheme is the same as the in-culture exposure, shown in Fig. 2.

### Solid Phase Extraction Desalter

**Fig 3:**
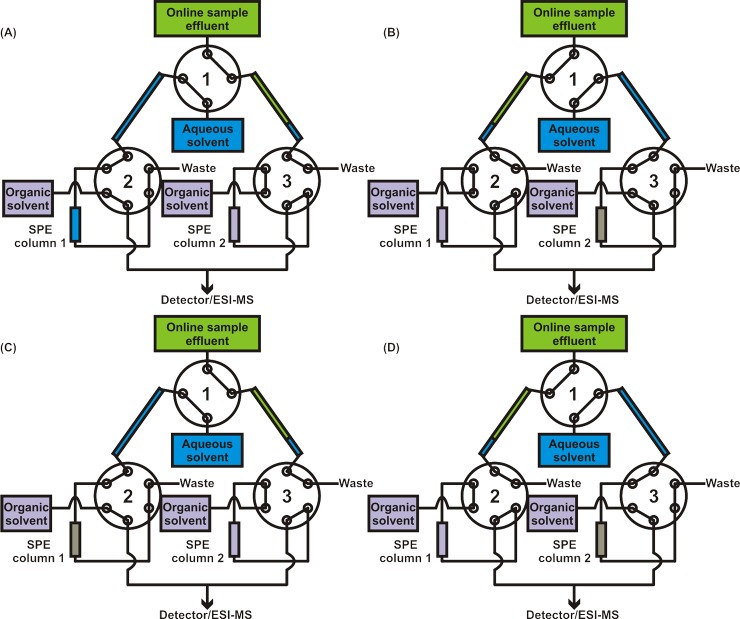
Solid phase extraction desalter. Setup starting from initial sample effluent flow incorporates two sample loops, three valves, and two C18 columns. During (A), sample effluent fills sample loop 2 for 9 minutes, while the aqueous solvent flows through sample loop 1, over column 1, and to waste. The organic solvent flows over column 2 and to the mass spectrometer. (B) Upon switching of the valves, the sample effluent fills sample loop 1 for 9 minutes. Aqueous solvent forces the 1.5 μL head of aqueous solvent, the 4.5 μL of sample effluent, and an additional 2.1 μL of aqueous solvent over column 2 to equilibrate the column, load the sample, and rinse away the salts. Organic solvent runs through column 1 and to the detector. (C) The next valve switch again exchanges the sample loop filled by effluent, while column 1 is equilibrated, loaded, and rinsed. The analytes captured on column 2 are eluted by the organic solvent and sent to the detector. (D) When the valves switch again, the sample effluent fills the opposite loop, column 2 is equilibrated, loaded, and rinsed, and column 1 is eluted with organic solvents and those analytes are sent to the detector. The pattern repeats until the experiment is completed, with each cycle requiring 9 minutes.

Columns were made of 360 μm OD/100 μm ID fused silica tubing and bomb-loaded in house with 3 μm, 300 Å, C18 phase Jupiter Bulk Packing (Phenomenex, Torrance, CA) using a PIP-500 Pressure Injection System (New Objective, Woburn, MA). Three 10-port Nanovolume UPLC Valves with 360 μm fittings, C72MH-4690ED (VICI Valco Instruments Co. Inc., Houston, TX) were used for the valve arrangement (Fig. 3). The aqueous solvent and both organic solvent lines, running at 500 nL/min, were supplied with an Eksigent Nanoflow Metering System (AB SCIEX, Framingham, MA), which has four independent flow channels. The output lines from the two downstream valves were connected with a micro-T junction and fed directly into the mass spectrometer via a nanoelectrospray ionization (nESI) source. Cheminert 360 μm unions (with 100 μm bore) were used for all tubing-to-tubing connections (not shown). Only fittings for 360 μm OD tubing were used, as the more popular 1/16” fittings, which require sleeves to connect to smaller bore tubing, resulted in leakage at high backpressures. Other than the columns, which were made of fused silica, 360 μm/50 μm PEEK tubing was used. Prior to experimental use a 2 μg/mL solution of polyarginine was run through the system to bind all non-reversible interaction sites. Elution cycles were then run overnight to ensure that all reversibly bound material was removed before experimentation. Sample loops were added to the system (as shown in Fig. 3) to reduce backpressure buildup.

### Online Cell Effluent Desalting and Mass Spectrometry Analysis

All online cell effluent experiments were conducted using PDMS MTNP devices. Cellular effluent from the device was processed online prior to mass spectrometry analysis using the online SPE desalter in Fig. 3. Cell effluents, driven by syringe pumps upstream of the bioreactor, were filtered with an inline filter (1 μm stainless steel frit, followed by a 0.5 μm polymer mesh, IDEX Health & Science, Oak Harbor, WA) and loaded into each sample loop. Sample loops, which were made of 360 μm OD/250 μm ID tubing, were 12.2 cm long, providing a sample loop volume of 6 μL. The continuous sample stream was diverted into each sample loop for exactly 9 minutes at 500 nL/min, thus filling the sample loop to 75% capacity. Because water was always flowing through these sample loops immediately prior to sample flow, a plug of 1.5 μL of water preceded each sample effluent plug. This plug served to quickly and roughly equilibrate the column with an aqueous solvent. Once the loop was filled to 75% with online sample effluent, the small water plug and sample effluent were passed over the column, using the aqueous solvent line to generate the necessary backpressure. Once the effluent had cleared the sample loop and had been entirely passed over the column, an additional 2.3 minutes or 2.1 μL of aqueous solvent (H_2_O with 0.1% formic acid) was run over the column to serve as the rinsing/purging step to remove residual salts. Following the salt purge, the column was eluted with organic solvent (90% methanol, 10% H_2_O, 0.1% formic acid). Each step of this process from the initial effluent flow through the SPE desalter is illustrated in Fig. 3. Data were collected using MassLynx software (Waters Corp., Milford, MA) by Data Dependent Analysis method cycle files, where each column elution was collected as an individual file. Collecting each column elution as individual data files aided sample analysis, as explained later.

### Data Processing and Multivariate Statistical Analysis

Resulting data sets from both online and in-culture experiments were processed using Waters MarkerLynx software along with Umetrics Extended Statistics software for multivariate statistical analysis (Waters Corp., Milford, MA). All spectra were corrected to sodiated HEPES buffer ([M+Na]^+^ exact mass 261.0888) and centroided, and peaks were normalized to 10,000 counts per sample. Spectra from samples analyzed through UPLC were deisotoped and underwent chromatographic peak detection. Data from online experiments were processed by a combined scan range. An intensity threshold was set for all data at 1000. Principal component analysis with Pareto scaling was performed to verify initial sample grouping. Further statistical analysis with orthogonal partial least squares-discriminate analysis was performed to identify significant contributors for group separation. Significance in abundance of exometabolomic species was determined through a Welch’s unpaired t-test using conservative confidence levels less than 0.05.

## Results

### Platform Integration and Evaluation

**Fig 4:**
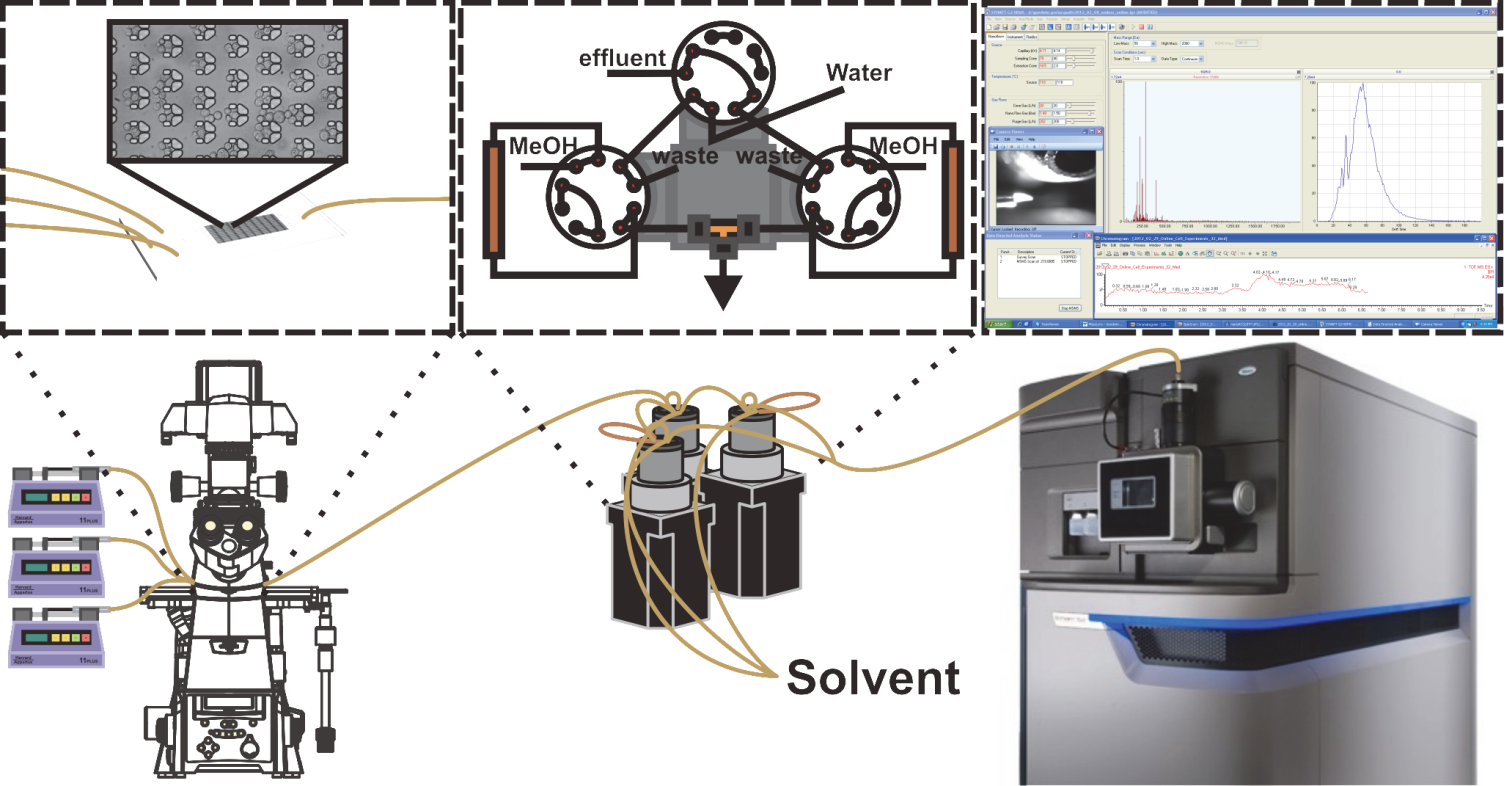
Multitrap Nanophysiometer (MTNP)-solid phase extraction (SPE)-nanoelectrospray ionization (nESI)-ion mobility (IM)-mass spectrometry (MS) platform. The platform includes Harvard Apparatus syringe pumps, a Nikon Eclipse Ti-e inverted fluorescence microscope with stage incubator, a solid phase extraction desalter with Eksigent and nanoAcquity pumps for solvent flow control, and a nanoelectrospray ionization source for continuous flow sample analysis with the Waters Synapt G2 ion mobility-mass spectrometer. Beyond the initial experimental setup, all components are fully automated and capable of running multi-hour experiments limited by the lifetime of cells in the MTNP.

Successful integration of the platform has been achieved, as shown in Fig. 4. The microfluidic bioreactor (MTNP) is controlled upstream by Harvard Apparatus syringe pumps, viewed under the Nikon Eclipse Ti-e microscope for optional fluorescent and brightfield imaging acquisition, and outfitted with a stage incubator for control of temperature, gas, and humidity. The effluent exiting from the MTNP flows through two inline filters for catching cell debris (1 μm stainless steel frit, followed by a 0.5 μm polymer mesh, IDEX Health & Science, Oak Harbor, WA). Once through the filters, the effluent fills one of the two sample loops vented to open air to avoid high backpressures in series with the compliant microfluidic device. After the effluent undergoes salt removal by the solid phase extraction desalting system, it is directed into the nESI source and sprayed into the nESI-IM-MS.

Initial studies, which paired a previous arrangement of the online SPE design [22] with MTNPs, resulted in ruptured devices. When LC pumps were used to rinse and elute the SPE columns, pressure would build up behind the column. When the valve was switched so that the MTNP was directly in series with the column, this high backpressure (>200 psi), would cause massive flow reversal and induce the PDMS device to physically delaminate from the glass to which it was bonded. This was alleviated by altering the valve arrangement to incorporate pressure-eliminating sample loops. By cutting off the direct pathway between the columns and the microfluidics, the buildup of pressure that occurred during rinsing/eluting was no longer in series with the compliant microfluidic device and would instead be vented to the waste port, as shown in Fig. 3. Although in extreme cases the inline filters downstream of the microfluidic device may become clogged with cells, thus interfering with effluent flows, this is a rare occurrence and can be prevented by using new filters for each experiment in addition to open-outlet cell loading (allowing cells to exit the device during loading before attaching to the downstream components).

The sample loop addition to the valve arrangement allowed for a two-step valve configuration, a simplification of the previous version. The new two-step arrangement generated a saw-toothed pattern of analyte elution as opposed to the rise-and-fall delta-function pattern observed with previous arrangements [22]. In this 180-minute experiment, an 18-minute-long cycle was used, producing an elution peak every 9 minutes. The sample loop volume was designed specifically to hold 9 minutes of sample effluent (4.5 μL at 500 nL/min flow rate). This cycle duration was determined to be optimal based on analyte concentrations. The file acquisition was set to account for the delay time from the device to the nESI source such that one column elution was captured per file while accounting for the roughly 12-second software delay between file acquisitions. Pump switching for control of the MTNP perfusion media was also timed with the SPE desalter valve switching.

Removal of salts in an online manner greatly increases signal-to-noise ratio. Yet with the low number of cells and high concentration of media components, detecting low concentrations of analytes is challenging. The signals are also affected by the dynamic range (∼10^5^) of the mass spectrometer [29]. Presumably, post-desalting, and given cellular utilization of nutrients in the media, HEPES at 10 mM is the analyte of greatest concentration. The high concentration of this species limits the lower range of detection of exometabolomic contents to roughly five orders of magnitude lower than HEPES. This HEPES concentration can be reduced if necessary, but as its purpose is to buffer the media to maintain a physiological pH, there could perhaps eventually be a tradeoff. When considering the small volumes associated with this platform, HEPES at 10mM would equate to 4.5 x 10^–8^ moles per filling of a sample loop. This would indicate that the dynamic range would allow for the detection of species as low as 10^–13^ moles per filling of the sample loop, or roughly 100nM. While detection of low concentration species remains a possible challenge, the ongoing advances in mass spectrometry technology will increase the detection capabilities of this system.

PDMS Surface Passivation for Increased Signal-to-Noise Ratio

While insulin is not necessarily a prime target of these experiments, it serves as an example of the potential complications from non-specific adsorption. Though high sensitivity is characteristic of mass spectrometry, our system seeks to identify secreted molecules from roughly 10^5^ cells. Detecting these low-level signals becomes a greater challenge when a portion of the signal is lost due to interactions with seemingly inert materials. Although the PDMS passivation schemes returned positive results (data provided in Supporting Information, S1 File, S2 Fig.), some metabolite, peptide, or protein species, such as insulin, are particularly “sticky” to most polymers and glass. In testing the capabilities of the system, we have noticed drastic reduction in and even absence of signal from insulin standards over time, even at low temperatures. Additionally, insulin hysteresis in the combined platform has been discovered after as long as 4 days of continuous perfusion of the SPE desalter tubing and columns. While the columns may be a source of insulin retention, this particular hysteresis occurred with freshly made columns, pointing to an alternate source of contamination that resulted in memory effects. The remaining sources of contamination could be from insulin retention in the PEEK and/or fused silica tubing lines, the valve rotors, or even on the source block or cone of the mass spectrometer. Further efforts for overcoming or reducing memory effects could include passivation of all components of the system or avoiding use of certain materials known to interact more readily with biological materials. Although analyte interaction with materials, PDMS in particular, is unavoidable to some extent, surface passivation provides a means of vastly minimizing the effect.

Comparison of UPLC-ESI-IM-MS to MTNP-SPE-nESI-IM-MS

One of the major technical challenges of organs-on-chips is the small fluid volumes available for analysis [16,17]. A significant reduction in time and the avoidance of handling and storage of small fluid samples are among the benefits of continuous sample flow from the microfluidic bioreactor to the solid phase extraction desalter and into the nESI-IM-MS. This integrated platform allows for the setup (∼2–3 hours), execution (∼4–8 hours), and data collection (no additional time) in the course of a day. Traditional in-culture experiments with UPLC-ESI-IM-MS analysis require possibly less initial setup (∼1 hour), roughly the same execution time (∼4–8 hours), and significant additional sample processing time (∼15–20 hours including overnight sample evaporation) and data collection (∼50 hours for 120 samples with a 25-minute UPLC time per sample), for a total of about 4 days until data are ready for processing, compared to our platform’s essentially real-time capability with a 9-minute sampling interval and in-line sample processing.

This suggests a major advantage of the integrated platform compared to in-culture experiments: the ease of obtaining mass spectra at multiple time points. Our process is automated with constant media perfusion control timed with the switching of the SPE desalter valves as well as the data file collection timing. With the in-culture experiments, a lengthier process is required to collect a single time point. The cell suspension must be centrifuged for 2 minutes, the supernatant removed, and the cell pellet resuspended in new media. The length of time required for this media change is on the same order of the time points taken with the integrated platform. Repeated centrifuging and pellet aspiration can introduce unnecessary stress to the cells, which may affect the cellular exometabolome and lead to profiles resulting from both the media exposure and additional cell stress.

One downside compared to liquid chromatography data is the absence of retention time information. While the solid phase extraction desalter uses a column similar to those found in an LC system, any slight timing discrepancy from one file collection to the next prevents the use of any retention time data. Confounding this issue is the fact that processing data within the Waters Masslynx software without retention time data available prevents the option for the removal of isotopes from the data. While this is a hindrance in some respects (roughly 3 times more peaks, redundant data, etc.), it can prevent the accidental removal of isobaric peaks of interest from the study. Since our platform is adaptable, integrating a chromatographic separation could be easily accomplished in a number of ways. Gradient elution could be utilized to achieve this type of separation without any additional hardware or software. An additional column could be utilized as part of the solid phase extraction desalter setup for further chromatographic separation as well, thus achieving all the benefits of liquid chromatography if desirable for a specific experimental setup.

### Cocaine Metabolism in Naïve and Experienced T Cells

Online Cellular Analysis

**Fig 5:**
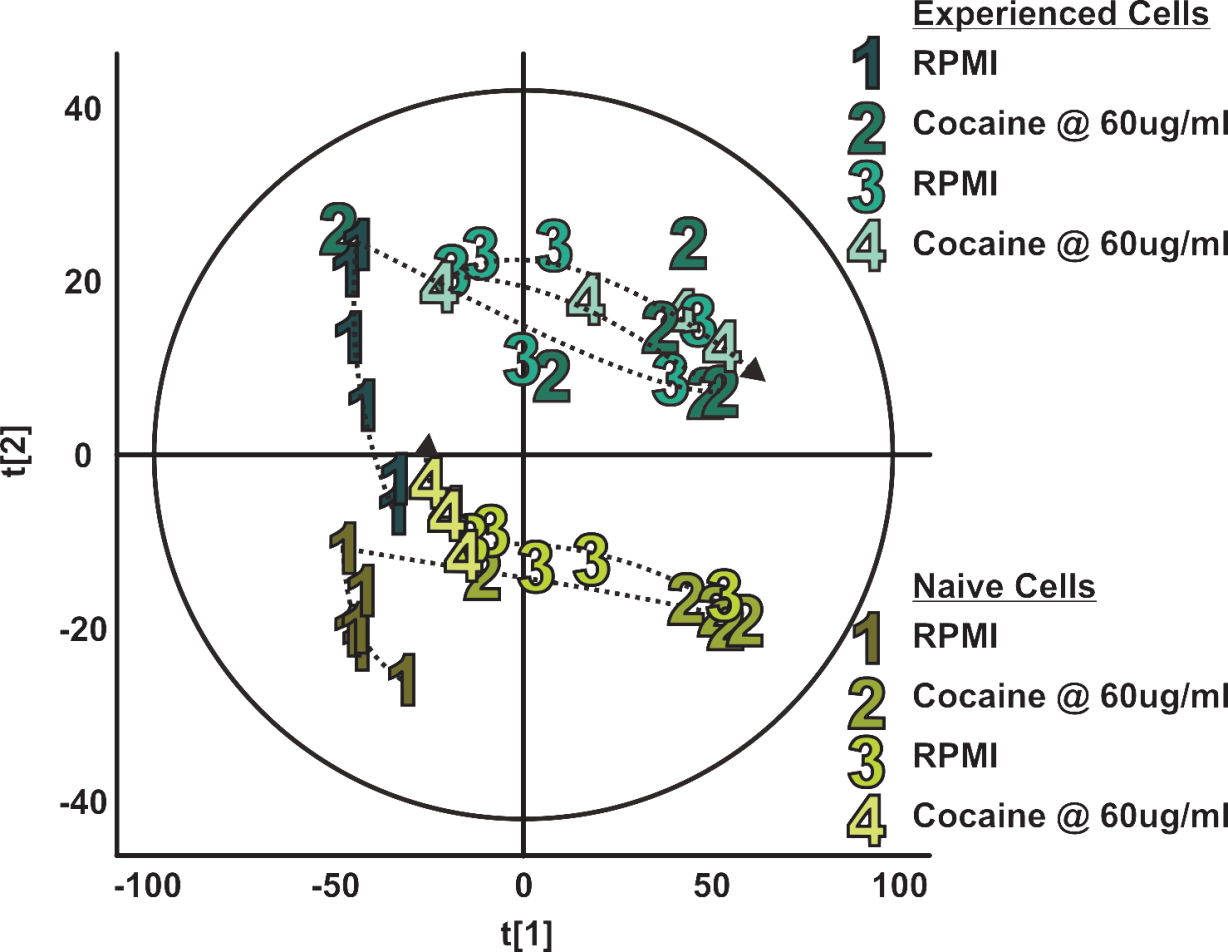
Exometabolomic profiles of naïve and cocaine-experienced Jurkat T cells from online cellular analysis. Walking principal component analyses of exometabolomic profiles of naïve (shades of yellow) and cocaine-experienced (shades of turquoise) Jurkat T cells were constructed following online cellular analysis. Each numerically labeled data point represents a 9-minute column elution, with six data points collected per step (all marked with the step number and connected in order of collection with the dotted line). In steps 1 and 3, both cell populations received plain RPMI. In steps 2 and 4, both received cocaine in RPMI at 60 μg/mL. As profiles switch from RPMI to cocaine exposure, the data points move towards the right and vice versa, with the exception of naïve cell step 4, which stays closer to step 1. Further analysis of the data reveals this inconsistency may be explained by the death of the cells. Data were grouped not only based on the experimental step, but also by the experience level of the cells, as the cells receiving a 4-hour pre-incubation in cocaine group separately from those that did not receive this dose.

Two populations of Jurkat T cells were compared in this study: naïve T cells that had never been exposed to cocaine and experienced T cells that had been incubating in cocaine at 60 μg/mL in RPMI 1640 for 4 hours prior to online experimentation. As shown in Fig. 5, a high degree of variance was observed based not only on what type of media was present in the bioreactor (*i*.*e*., plain RPMI media or cocaine RPMI media), but also whether the cells experienced cocaine earlier in the day (*i*.*e*., whether the cells were experienced or naïve to cocaine exposure). The major unique contributors to group separation between naïve and experienced exometabolomic profiles included m/z 283, m/z 187, m/z 399, m/z 157, and m/z 337 (all at elevated abundance in the experienced group compared to the naïve group). The metabolites contributing to group separation between type of media to which cells were exposed in the MTNP consisted of m/z 312, m/z 182, m/z 304, m/z 290, and m/z 150. Though the data analysis pipeline prevented removal of isotopes, any m/z present in the list of top contributors negated the inclusion of their respective isotopes from these lists.

**Fig 6:**
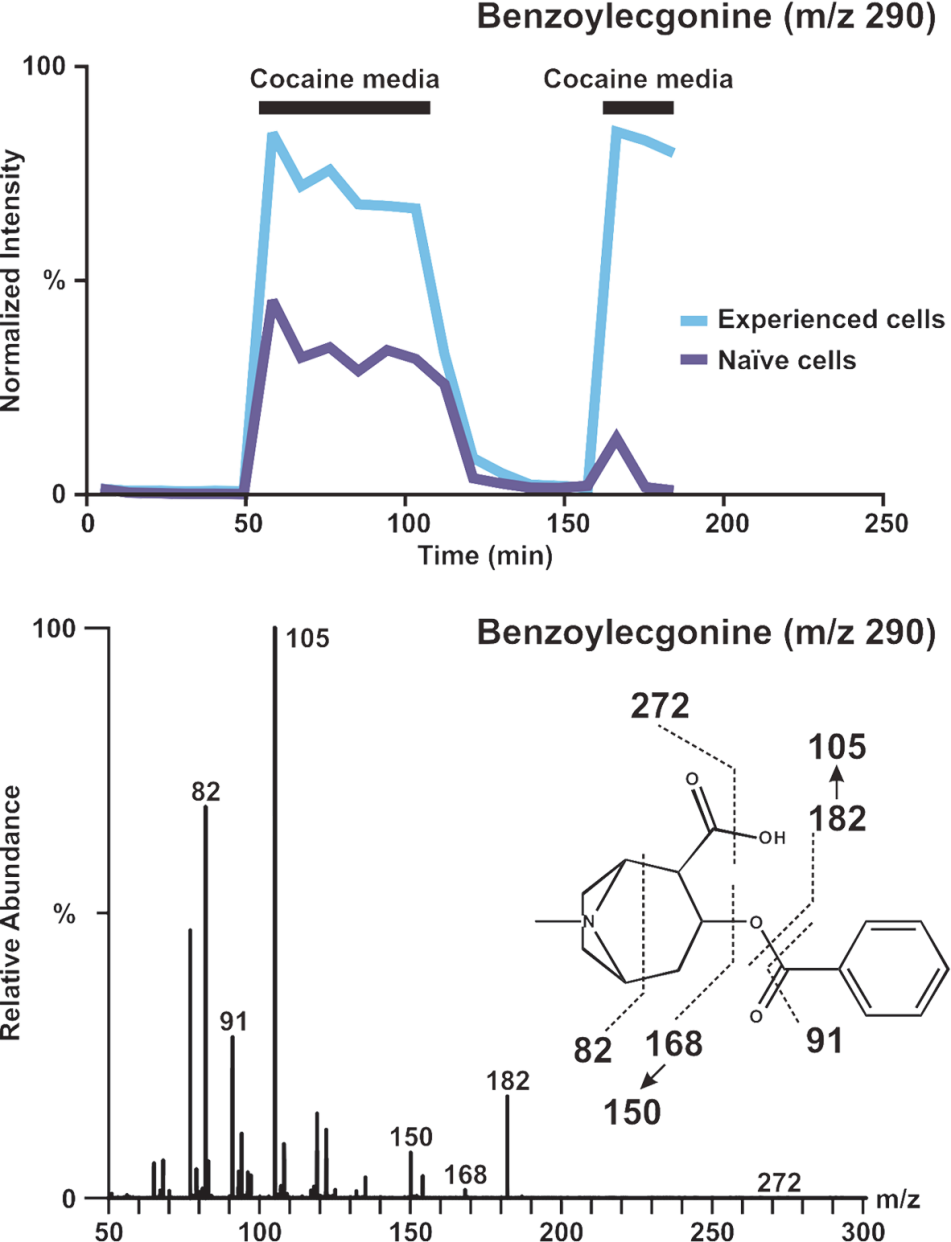
Benzoylecgonine (BE) time course and fragmentation data. Top: BE time course data for experienced (blue) and naïve (purple) cells. While data were gathered sequentially, plots are overlaid to highlight the increased abundance of BE in experienced cells. The absence of the expected increase in BE corresponding to step 4 (the last step of cocaine exposure) suggests a decrease in cocaine metabolism, possibly due to cell death. Bottom: The fragmentation spectra of BE are shown with parent ion of m/z 290.

**Fig 7:**
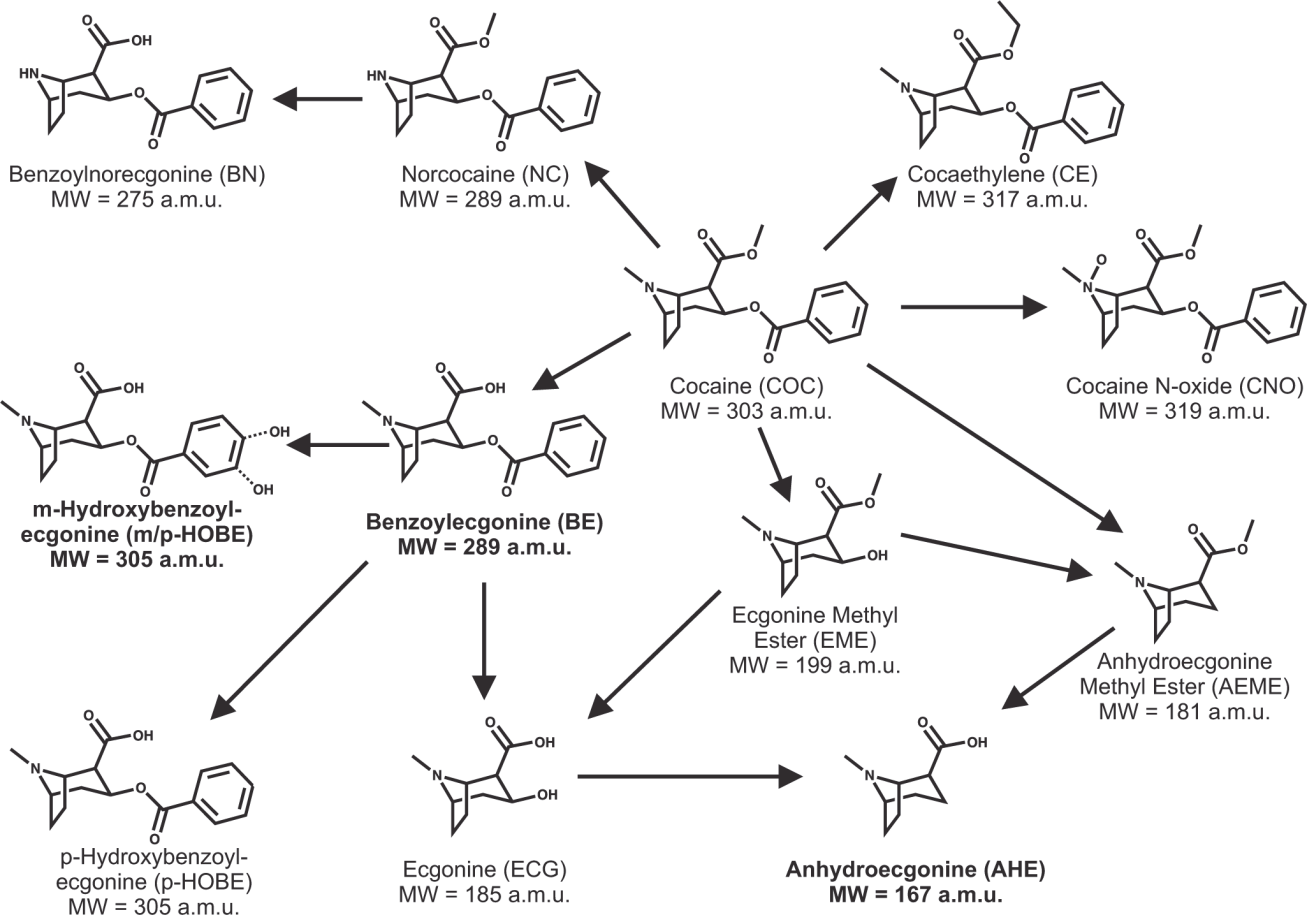
Metabolism of cocaine showing molecular weight for each metabolite. The linkages in this network were adapted from Xia *et al*. [30]. Putatively identified species are shown in bold.

Benzoylecgonine (BE) (m/z 290 as [M+H]^+^, m/z 312 as [M+Na]^+^), a primary metabolite of cocaine, was identified as a contributing factor to the separation between the cocaine exposure steps and plain RPMI steps. This metabolite additionally contributed to the separation of populations of naïve cells and experienced cells. Analysis of this metabolite over the time course of the experiment revealed an expected increase during cocaine exposure steps, but also showed a significant increase from naïve to experienced cells with a very conservative *p-value of 5*.*7 x 10* ^*–4*^ (Fig. 6). Expected levels of BE produced by degradation or metabolism of cocaine in the second cocaine exposure of the naïve cell population are notably absent. Further analysis of this apparent reduction in BE level during this exposure period revealed the likelihood of cellular death as a contributor to this result. Fragmentation spectra revealed fragment ions m/z 82, m/z 91, m/z 105, m/z 150, m/z 168, m/z 182 and m/z 272, as shown in Fig. 6. Analysis of the remaining top three contributors to separation based on media revealed cocaine at m/z 304, anhydroecgonine methyl ester (AEME) at m/z 182 (produced from dehydration of ecgonine methyl ester (EME) rather than the pyrolysis of cocaine), and ecgonine aldehyde, the decomposition product of EME, at m/z 150. Cocaine metabolic pathways are described in Fig. 7.

To verify that this increased BE abundance was not purely a result of non-enzymatic hydrolysis of cocaine to BE in aqueous solutions over the time course of the experiment, we omitted the Jurkat cells from an experiment conducted simultaneously with those for the naïve and experienced cells. The cell-free media, either with or without cocaine, depending upon interval in the protocol, was sampled and analyzed in the same manner as the media conditioned by the cells. In order to compare the experiment with cells to those without cells, we normalized the BE intensity to the cocaine intensity. On average, the percent of the total normalized BE created by non-enzymatic hydrolysis of cocaine was 34.1% in step 2 of the naïve cell experiment, while the corresponding percentage for step 2 of the experienced cell experiment was 34.5%. There may be other not-yet-identified mechanisms for the breakdown of both cocaine and BE, possibly involving processes shown in Fig. 7. Hence the cells contribute to no more than ∼66% of the BE reported in Fig. 6. The statistical significance of the differences between naïve and experienced BE production is not affected by this correction.

**Fig 8:**
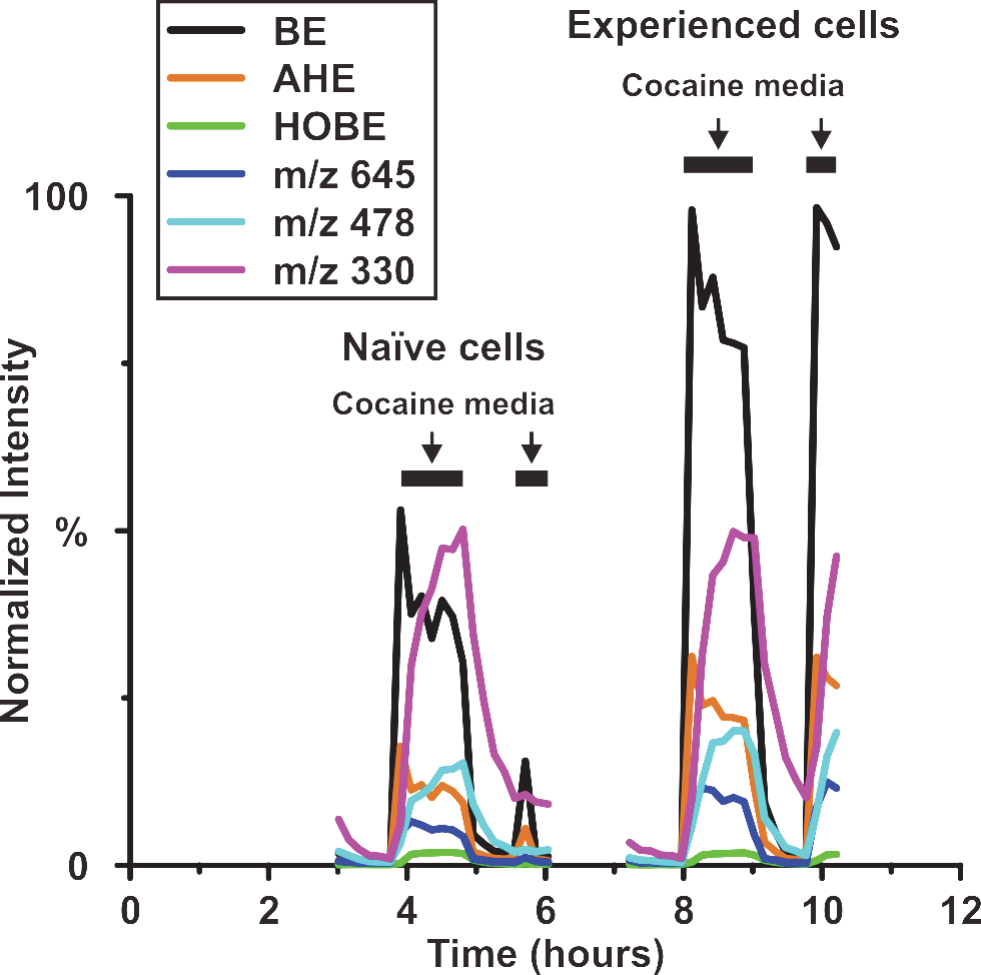
Additional metabolite time course data compared to benzoylecgonine (BE). Experimental conditions for each group of cells are shown above the graph with solid black lines indicating exposure to cocaine media. Anhydroecgonine (AHE) and hydroxybenzoylecgonine (HOBE), two additional metabolites of cocaine, provide examples of both variation between naïve and experienced cell groups in the case of AHE and consistency between these two groups in the case of HOBE. The increases in BE and AHE from naïve to experienced groups are statistically significant with respective *p values* of *5*.*7 x 10* ^*–4*^ and *1*.*12 x 10* ^*–3*^. Three unidentified metabolites (m/z 645, m/z 478, and m/z 330) that contribute to the separation between media exposure groups are also shown. m/z 330 and m/z 478 show no statistical significance between naïve and experienced cell groups while the increase in m/z 645 is statistically significant (*p = 1*.*6 x 10* ^*–3*^ *)*.

The time course of additional metabolites is provided in Fig. 8, including cocaine metabolites anhydroecgonine (AHE) (m/z 168) and hydroxybenzoylecgonine (HOBE) (m/z 306) (shown in bold in Fig. 7), as well as several unknown metabolites (m/z 330, m/z 475, m/z 678). Some of these additional metabolites have higher abundance in the cocaine-experienced population while others have no overall change in abundance. Overall, BE, AHE, and m/z 645 show significant increases from naïve to experienced cell population (*p-values = 5*.*7 x 10* ^*–4*^, *1*.*12 x 10* ^*–3*^, *and 1*.*60 x 10* ^*–3*^, respectively). While AHE is a typical product of AEME (the pyrolysis product of cocaine), some reports have shown that the metabolic pathway from cocaine into AEME and AHE could result from loss of water of EME or ecgonine [30]. While there is much evidence that AEME and AHE can form as a result of the analysis technique, this is typical of gas chromatography separations that require vaporization of compounds, thus risking alteration of thermolabile compounds such as cocaine and its metabolites [31]. Electrospray ionization is utilized when this type of compound is under investigation.

In-Culture Cellular Analysis

**Fig 9:**
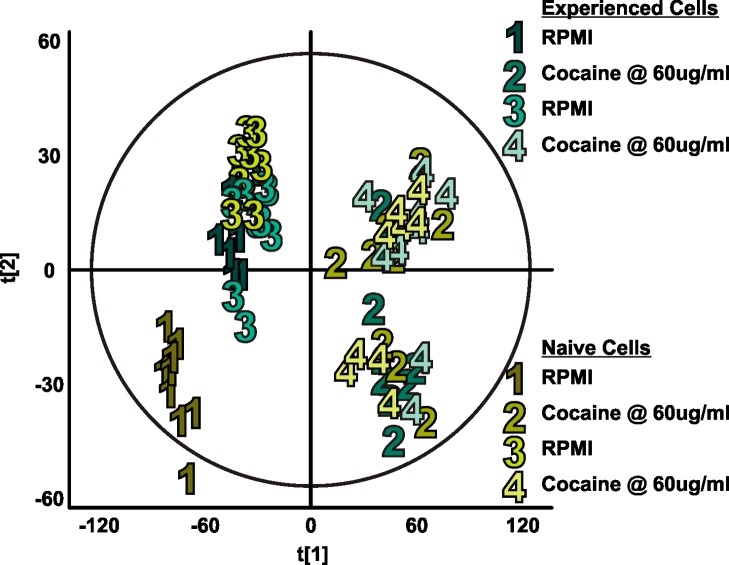
Exometabolomic profiles of naïve and cocaine-experienced Jurkat T cells from in-culture analysis. Walking principal component analysis of exometabolomic profiles of naïve (shades of yellow) and cocaine-experienced (shades of turquoise) Jurkat T cells using in-culture analysis. Each numerically labeled data point represents the end point of a 54-minute exposure to the indicated media. As with the online experiment, during steps 1 and 3 both cell populations received plain RPMI, while during steps 2 and 4 both received cocaine in RPMI at 60 μg/mL. Variance between steps 1 and 3 and steps 2 and 4 results in unsupervised grouping according to media exposure type. Separation between naïve and experienced cell groups, particularly when under cocaine exposure, does not occur to the same degree, as yellow and turquoise points corresponding to steps 2 and 4 are heavily intermixed.

To compare this instrumentation platform, as well as the resulting biological data of naïve and cocaine-experienced T cells, with the current standard in mass spectrometry analysis of biological samples, we replicated the online experiment in culture using UPLC-ESI-IM-MS. Fig. 9 shows the principal component analysis plot demonstrating sufficient variance when comparing steps 1 and 3 (plain RPMI 1640 exposure) with steps 2 and 4 (cocaine exposure). In the online experiment, we are able to see separation between cocaine-experienced cells and naïve cells, a separation that is absent from the in-culture study, with the exception of the initial RPMI exposure of the naïve cell populations. While one major difference is the number of time points per step of media exposure, as discussed previously, replicating the 9-minute time point sampling of the online system would confound the length of time needed for media switching, as well as inflict unnecessary stress on the cells.

## Conclusions

### Platform Capabilities

We have described the integration, workflow, and proof of concept of a technology platform for near real-time detection of the dynamic cellular exometabolome based on the combination of a microfluidic bioreactor, an online SPE desalting arrangement, and mass spectrometry. A great strength of this platform is its adaptability to different cell types and experimental conditions. Microfluidic cell trapping devices can be customized to the size of any adherent or non-adherent cell type, and they provide a solution to the dilution issues found in traditional well plate experiments. In this work, we demonstrate cell trapping and experimentation on naïve and cocaine-experienced Jurkat T cells. While this work shows only one model system based around cocaine exposure, the environmental stimuli are limited only by the number of pumps one has available for providing variable perfusion conditions and the temperature change and gas exchange rates an incubator is capable of generating. Studies are under way to incorporate low-cost micropumps commensurate with a microfluidic platform, such as those reported by Darby *et al*. [32]. Thus, this platform is directly amenable to exposure of cells to other environmental drugs or toxins. The online SPE desalting arrangement allowed for sufficient desalting to permit a temporal resolution of 9 minutes. While this temporal resolution is not by any means a significant advance for cellular chemical detection methods, because it is dictated by the analyte abundance and detection capabilities of the time-of-flight mass analyzer, it is (depending on the mass analyzer) the best resolution possible for this biological system using mass spectrometry. Because it is trivial to scale the loading time in this arrangement based on detection power, we believe a technology platform of this general system will be of considerable utility to the biological community, particularly as mass analysis detectors improve in the coming years.

### Cellular Memory of Cocaine Experience

Cocaine metabolism in naïve and experienced Jurkat T cells was investigated with this near real-time platform developed for the study of the cellular exometabolome. While it is well known that cocaine has an effect on immune cells, there has been no prior demonstration (though the idea has been suggested [33]) of even a short-term immune cell memory of prior cocaine exposure. With the advent of this innovative online platform, unique metabolic signatures (Fig. 5) are obtained that are absent from the “in-culture” data (Fig. 9) or perhaps lost due to the increased sample processing and UPLC analysis time. A concentration of cocaine higher than typically found in circulation was applied to cells to ensure that a cellular response was achieved for purposes of platform validation, not for modeling the *in vivo* conditions. Upregulation of cocaine metabolism into benzoylecgonine in experienced cells demonstrates one contributor to the unique exometabolomic profile resulting from previous cocaine experience. Anhydroecgonine, as well as unknown metabolites m/z 645 and m/z 478, are also upregulated in cell populations with prior cocaine exposure, leading to the possibility of indicators of immune cell memory of cocaine other than cocaine metabolites alone.

While there is a previously reported non-enzymatic degradation rate of cocaine into benzoylecgonine at physiological temperatures and pH [34], we were able to confirm a rate specific to this platform. Through comparison of the BE to cocaine ratios from naïve and experienced cell experiments, as well as the platform absent of cells, it is evident that the portion of BE abundance from non-enzymatic degradation does not entirely explain the significant increase in BE during the cocaine exposure steps in the experienced cells, indicating that the response is due to a unique exometabolomic profile of T cells with prior cocaine exposure. Further analysis of cellular memory of cocaine exposure, in particular at a range of concentrations, is warranted based upon these findings.

## Supporting Information

S1 Fig PDMS silanization scheme. Hydrolysis of methoxy group from PEGn trimethoxysilane causes the formation of silanol groups (a). PDMS activation by O_2_ plasma (b (top)), silane deposition (b (middle)), condensation of the silane into chains (b (bottom)), hydrogen bond formation between silanol and oxidized PDMS surface (c (left)), and covalent bond formation between silane and PDMS surface (c (right)) complete the silanization process.
(JPG)

S2 Fig Reduction of nonspecific adsorption by PDMS silanization. PDMS channels were perfused with FITC-insulin for 30 minutes (green), followed by 30 minutes of rinsing with Ringer’s solution (pink). The top three fluorescent images show the surface-bound insulin at 1 minute, 31 minute, and 60 minutes, with the bottom three images corresponding to the surface-modified channels. Silanized PDMS channels (blue line) retain (at maximum) only 25% of the total insulin retained by untreated PDMS (red line).
(JPG)

S1 File PDMS Surface Modification for Reduction of Non-Specific Adsorption. (PDF)
