# Supplementary figures and images for: Real-Time Cellular Exometabolome Analysis with a Microfluidic-Mass Spectrometry Platform
Source: PLoS One. 2015 Feb 27;10(2):e0117685. doi: 10.1371/journal.pone.0117685 (PMC4344306; doi:10.1371/journal.pone.0117685)

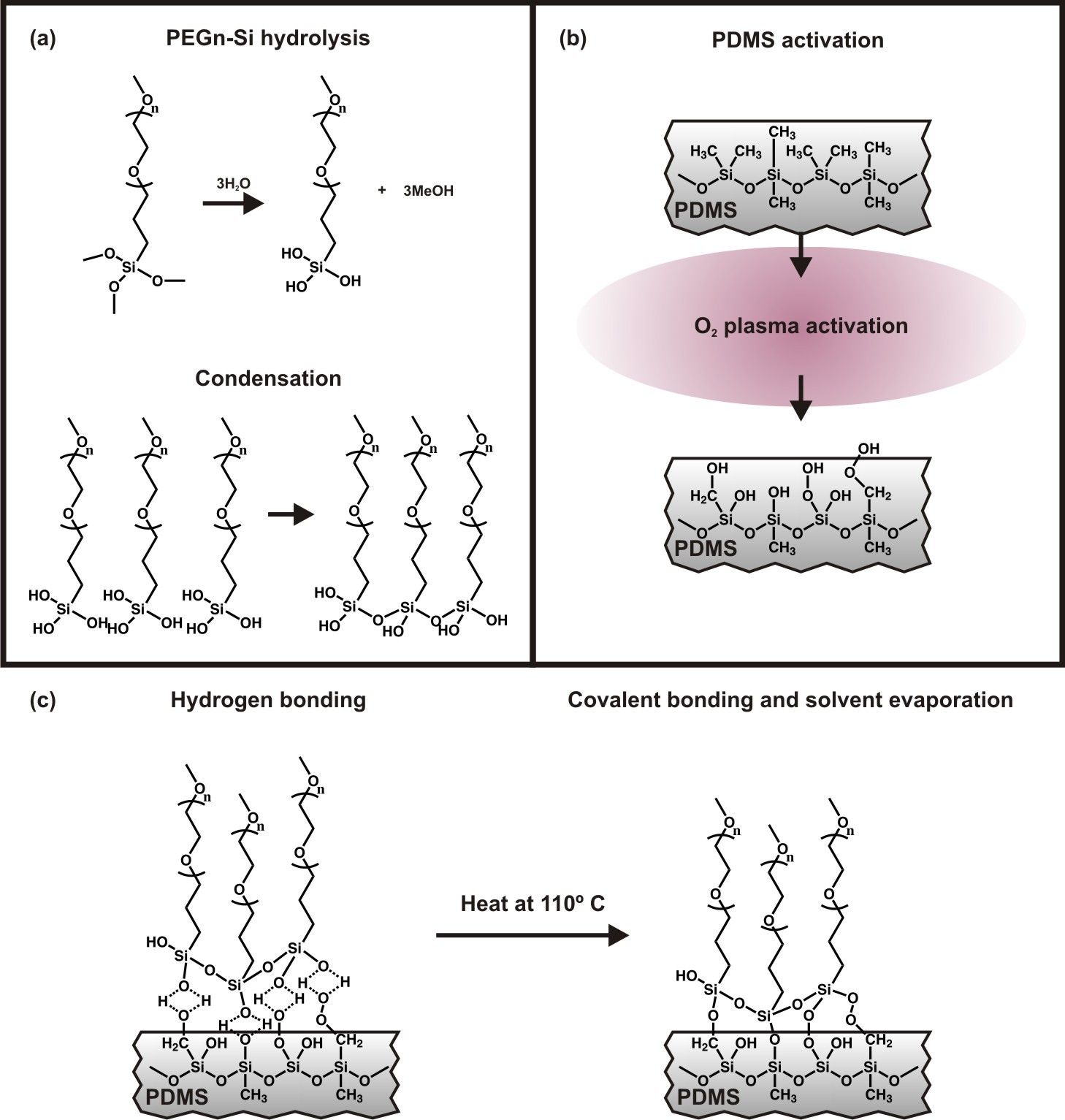

Supplement: S1 Fig — Hydrolysis of methoxy group from PEGn trimethoxysilane causes the formation of silanol groups (a). PDMS activation by O2 plasma (b (top)), silane deposition (b (middle)), condensation of the silane into chains (b (bottom)), hydrogen bond formation between silanol and oxidized PDMS surface (c (left)), and covalent bond formation between silane and PDMS surface (c (right)) complete the silanization process. (JPG) [file pone.0117685.s001.jpg]

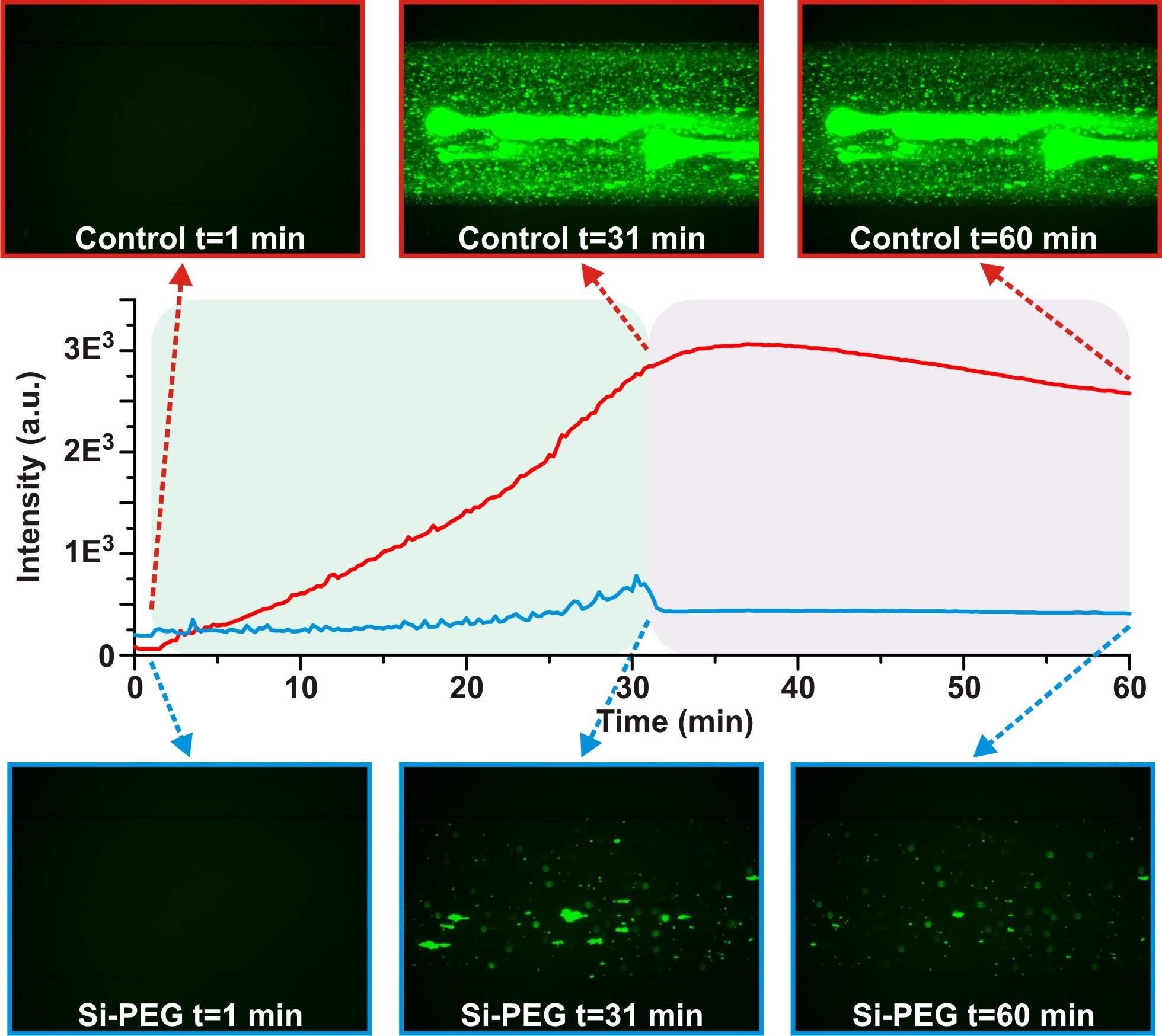

Supplement: S2 Fig — PDMS channels were perfused with FITC-insulin for 30 minutes (green), followed by 30 minutes of rinsing with Ringer’s solution (pink). The top three fluorescent images show the surface-bound insulin at 1 minute, 31 minute, and 60 minutes, with the bottom three images corresponding to the surface-modified channels. Silanized PDMS channels (blue line) retain (at maximum) only 25% of the total insulin retained by untreated PDMS (red line). (JPG) [file pone.0117685.s002.jpg]
