# Supplementary material for: Real-Time Cellular Exometabolome Analysis with a Microfluidic-Mass Spectrometry Platform
Source: PLoS One. 2015 Feb 27;10(2):e0117685. doi: 10.1371/journal.pone.0117685 (PMC4344306; doi:10.1371/journal.pone.0117685)
Supplement: S1 File — (PDF) [file pone.0117685.s003.pdf]

Real-Time Cellular Exometabolome Analysis with a Microfluidic-Mass Spectrometry Platform

Supporting Information

Christina C. Marasco, Jeffrey R. Enders, Kevin T. Seale, John A. McLean, John P. Wikswo, Vanderbilt University

PDMS Surface Modification for Reduction of Non-Specific Adsorption

The use of a biological model for testing the instrumentation platform does have a major limitation in the form of potential loss of metabolite signal due to adsorption to PDMS bioreactor surfaces. Rectification of these types of issues requires changing the microfluidic device material or modification of PDMS surface properties. Switching device materials, and thus the techniques for producing such devices, often requires substantial changes in infrastructure as well as loss of desirable PDMS properties such as optical clarity and small feature size. Surface modification is more plausible, as procedures can be added onto existing fabrication or experimental methods. A variety of types of surface modification of PDMS microfluidic devices have been investigated, such as chemical vapor deposition, sol-gel coating, silanization, surfactant coating and dynamic coating (reviewed elsewhere [1,2]). Due to the nature of this combined technology platform with mass spectrometry downstream of the microfluidic device, care must be exercised in the selection of a surface modification method. The presence of species in the bioreactor effluent necessary for the alteration of the PDMS surface can lead to complications in the detection of desired cell secretions due to issues of dynamic range. Instability of species used in surface modification can additionally lead to adsorption of secreted cellular materials to PDMS. A stable, inert coating is crucial for downstream mass spectrometry. Surfactants and dynamic coating methods are not appropriate for this application as the presence of electrostatically bound polymer or added analytes to the solution are not compliant with detecting low levels of cellular signals. One method leading to a covalently bound surface modifier is silanization. Though silanes are often deposited on surfaces through chemical vapor deposition and can result in the production of chlorine gas, ethoxy- and methoxy-silanes can be purchased in a liquid form, resulting in ease of application to PDMS surfaces for the prevention of non-specific adsorption.

PDMS Surface Modification

Liquid 2-[methoxy(polyethyleneoxy)$_{6\text{-}9}$propyl]trimethoxysilane (PEG-silane) was obtained from Gelest (Morrisville, PA). Immediately following plasma treatment, devices were intubated and a 2% solution of PEG-silane in 95% ethanol, 5% H_2O, and 11 mM acetic acid was injected until the device interior was filled. Devices were allowed to incubate at room temperature for 10 minutes followed by a rinse with ethanol, then water. A final incubation on a hot plate at 110°C for 10 minutes was then performed to evaporate any remaining solvent and complete the surface modification. Fig. S1 portrays the steps in the covalent surface modification of PDMS.

After at least 24 hours at room temperature, each device was perfused with fluorescently labeled insulin at a rate of 500 nL/min for 30 minutes and then rinsed with Ringer's buffer (118 mM NaCl, 5.4 mM KCl, 2.5 mM $CaCl_2$, 1.2 mM $MgSO_4$, 1.2 mM KH_2PO_4, 20 mM HEPES) for another 30 minutes. Images were collected every 15 seconds during the perfusion and rinsing stages with an inverted Nikon Eclipse Ti-e fitted with a 495

nm/521 nm excitation/emission filter set for fluorescent imaging of adsorbed insulin. Images were aligned and processed with Image J for comparison of total fluorescence intensity.

Reduction of Non-Specific Adsorption

Using fluorescently labeled insulin, the adsorption of insulin to the surface of PDMS could be visualized. Fig. S2 provides both pictorial and graphical representations of the adsorption of insulin to PDMS and PEG-Si-PDMS. During the 30 minutes of fluorescent insulin exposure, the intensity increases drastically for the control PDMS and less drastically for the silanized PDMS with a relative maxima of 4:1.

References

1. Zhou JW, Ellis AV, Voelcker NH (2010) Recent developments in PDMS surface modification for microfluidic devices. Electrophoresis 31: 2-16.

2. Wong I, Ho CM (2009) Surface molecular property modifications for poly(dimethylsiloxane) (PDMS) based microfluidic devices. Microfluid Nanofluid 7: 291-306.
